# Reproductive risks of microplastics in assisted reproduction

**DOI:** 10.1016/j.ebiom.2026.106426

**Published:** 2026-08-12

**Authors:** Di Mao, Wenhui Nan, Jizhou Li, Bingkai Su, Rong Li, Kai-Lun Hu, Xunsi Qin

**Affiliations:** a State Key Laboratory of Female Fertility Promotion, Center for Reproductive Medicine, Department of Obstetrics and Gynecology, National Clinical Research Center for Obstetrics and Gynecology, Peking University Third Hospital, Beijing 100191, China; b Key Laboratory of Assisted Reproduction (Peking University), Ministry of Education, Beijing 100191, China; c Beijing Key Laboratory of Collaborative Innovation in Frontier Technologies for Population Quality, Beijing 100191, China; d National Clinical Key Specialty Construction Program, P. R. China (2023), Beijing 100191, China

**Keywords:** Microplastics, Assisted reproductive technology, Fertility, Transgenerational effect

## Abstract

Microplastics (MPs) are widespread environmental contaminants detected in multiple human tissues, raising significant health concerns. The detection of MPs from human reproductive tissues largely indicates potential risks to reproductive health in both men and women. Although the effects of MPs on reproductive diseases remain poorly understood, MPs may be associated with ovarian dysfunction, endometrial disorders, and impaired sperm quality. Moreover, microplastic exposure may pose transgenerational risks through epigenetic reprogramming. Here, we draw attention to the potential threat of MP contamination to assisted reproductive technology (ART), where extensive use of plastic materials may expose gametes and embryos to MPs, potentially affecting embryo development and clinical outcomes. However, direct evidence that standard ART procedures elevate MP exposure or compromise clinical outcomes remains scarce, rendering the potential impact speculative. To elucidate and mitigate these risks, the field may require standardised detection protocols, rigorous contamination controls, prospective multicentre cohorts, and safer materials.

## Introduction

Microplastics (MPs, defined as plastic particles smaller than 5 mm, including nanosized plastics smaller than 1 μm) have emerged as novel environmental pollutants and have become a global public health issue in recent years.^1^ Research confirms that MPs are widely distributed across marine, freshwater, terrestrial, and atmospheric ecosystems, being universally detected in various environmental media samples.^2^^,^^3^ These particles have been detected worldwide and in numerous human tissues, attracting widespread attention. Existing research has revealed the health toxicity of MPs across multiple organs, including the brain, blood vessels, lungs, liver, kidneys, small intestine, placenta, and ovaries.^4^, ^5^, ^6^, ^7^, ^8^ Beyond direct exposure levels, MPs frequently act as vectors for other toxic substances, including heavy metals, persistent organic pollutants, pathogens, and even antibiotic resistance genes.^9^, ^10^, ^11^ Consequently, the severe impact of MPs on human health is progressively coming to light and commanding significant focus.

Reproductive health represents the starting point for safeguarding overall human health, fundamentally influencing individual fertility, family harmony, population security, and societal development. Infertility remains a persistent global reproductive health crisis that leads to profound societal and clinical consequences. Reports indicate that 1.8% of men and 3.7% of women suffer from infertility worldwide; furthermore, the prevalence of infertility has grown persistently over the past thirty years and is projected to continue rising over the next twenty years.^12^ Amid this current state of reproductive health, the impact of MP exposure has garnered heightened attention. Our previous research indicates that MPs may penetrate the uterine environment through vaginal exposure, underscoring a concerning new dimension in female reproductive health.^13^ Additionally, the recent identification of MPs in human penile tissue highlights the critical need for further investigation into how this pollution impacts the fertility and sexual health of both sexes.^14^ Collectively, these converging lines of evidence strongly suggest that MP exposure serves as a significant risk factor for infertility.

However, for patients experiencing infertility, undergoing assisted reproductive technology (ART) means they inevitably encounter increased opportunities for MP exposure throughout the clinical and laboratory processes. Consequently, this personal view aims to comprehensively summarise the current evidence regarding the potential risks of MPs within the context of ART. By systematically evaluating these factors, we hope to provide deeper insights and establish clear research directions for future investigations, ultimately offering essential warrants for clinical practice.

## Reproductive toxicity of MPs: physiological foundations and implications for ART

There is growing evidence indicating the presence of MPs in human reproductive organs and biological samples. We conducted a scoping review using the terms “microplastics”, “nanoplastics”, “semen”, “follicular fluid”, “testis”, “ovary”, “endometrium”, and “uterine” in the Medline electronic database without restrictions on language or publication year. All studies providing evidence of the presence of MPs in human reproductive systems are summarised in Table 1. We identified 23 studies, the majority of which were conducted in Chinese populations (17/23), while two were conducted in Italy, one in Mexico, one in Türkiye, and one in Malaysia. Detection methods included pyrolysis gas chromatography mass spectrometry (Py-GC/MS), laser direct infrared imaging (LDIR), Fourier transform infrared spectroscopy (FTIR), optical photothermal infrared, Raman spectroscopy, and scanning electron microscopy (SEM). Detection rates were 60–100% in semen, reached 100% in testicular tissue, ranged from 21.9–100% in follicular fluid (FF), and 65–100% in endometrial tissues.

**Table 1:** Characteristics and main findings of studies detecting MPs in the human male and female reproductive systems.

Study	Location	Samples	Detection methods	Main findings
Male reproductive system				
Zhou et al., 2023^15^	China	6 testis, 30 semen	Py-GC/MS, LDIR	100% detection (semen: 0.23 particles/mL; testis: 11.6 particles/g); Predominant: PE/PVC (semen), PS (testis); Fragments were the primary shape in testis.
Montano et al., 2023^16^	Italy	10 semen	Raman	60% detection; Predominant: PP, PE, PET, PS, PVC, PC.
Hu et al., 2024^17^	Mexico	23 testis	Py-GC/MS	100% detection (mean 328.44 μg/g); Predominant: PE; Negative correlation observed between PVC/PET levels and normalised testis weight.
Li et al., 2024^18^	China	40 semen	Raman	100% detection (mean 2 particles/sample); Predominant: PS (31%); PS exposure linked to relatively higher sperm motility compared to PVC; no significant association with morphology.
Zhang et al., 2024^19^	China	113 semen	Raman	100% detection; Predominant: PS, PP, PE; PTFE exposure significantly associated with decreased total sperm count, concentration, and progressive motility.
Guo et al., 2025^20^	China	45 semen	LDIR	75.6% detection (17.0 ± 42.0 particles/g); Predominant: PET, BR, CPE; PET exposure associated with notably reduced sperm progressive motility.
Kong et al. 2025^21^	China	51 semen, FF	Py-GC/MS	Detected in semen (PE 3.02 μg/g, PVC 2.67 μg/g) and FF (PE 1.21 μg/g, PVC 1.85 μg/g); Predominant: PE, PVC; Semen PVC linked to reduced motility; FF PE/PVC linked to lower IVF fertilisation rates.
Wang et al. 2025^22^	China	4 semen	Py-GC/MS	100% detection (PE levels up to 865.55 μg/mL); Predominant: PP, PE, PET.
Ilki et al., 2025	Türkiye	98 semen (50 varicocele, 48 Ctrl)	Light microscopy	84% detection in varicocele vs. 62.5% in controls (median 8 vs. 4 particles/mL); MP burden negatively correlated with sperm concentration, progressive motility, and normal morphology.
Su et al., 2025	China	Sperm specimens	O-PTIR	Identified sub-10-μm PS MPs with protein coronas in mechanically retrieved sperm; Scratching PS Petri dishes generated 100-22,000 particles/mL exposure dose.
Praveena et al., 2026	Malaysia	115 semen	Raman, SEM-EDX	100% detection (0.2–3.3 particles/mL); Predominant: PP (48%), PET (30%); Particles exhibited surface-associated elemental profiles including phosphorus, calcium, iron, and copper.
Ren et al. 2026^23^	China	6 semen	LDIR	100% detection (104 ± 53.58 particles/g); Predominant: CPE, PVC, PS, PE; MP concentration significantly higher in asthenozoospermia group vs. normal controls.
Female reproductive system				
Dong et al., 2024^24^	China	60 adnexa (adenomyosis, cysts, tubes)	micro-Raman	100% detection (1.5 ± 1.2 particles/g); Predominant: PE, PP, PE-co-PP; 70% of MPs were <20 μm (primarily fibres and fragments).
Qin et al., 2024^13^	China	22 endometrium	Raman, FTIR	100% detection (size 2–200 μm); Predominant: PA, PU, PET, PP, PS, PE.
Sun et al., 2024^25^	China	20 endometrium	LDIR	High abundance (median 21 particles/100 mg); Predominant: Ethylene-acrylic acid copolymer, PE; Higher MP exposure correlated with drinking habits and chewing gum.
He et al., 2025^26^	China	16 endometrial polyps, 14 Ctrl	Py-GC/MS, LDIR	MP abundance significantly higher in polyps vs. normal endometrium; Predominant: PMMA, ACR, PU, PBAT, EVA.
Montano et al., 2025^27^	Italy	18 FF	SEM-EDX	77.8% detection (mean 2191 particles/mL); Positively correlated with FSH levels; no correlation found with AMH or live birth rates.
Ni et al., 2025^28^	China	19 FF	Py-GC/MS, LDIR	21.9% of analysed particles in FF were identified as MPs; Predominant: PE, PVC, PP, PS.
Wang et al., 2025^29^	China	44 FF	Raman	86.4% detection rate (highest for PE); Predominant: PE; Negatively correlated with fertilisation rates.
Xu et al., 2025^30^	China	40 diseased and normal endometrium tissues in patients with UF, 40 myometrium tissues in healthy populations	Raman spectroscopy	Detection rates: 75% in diseased UF (2.63 ± 1.77 particles/g), 65% in normal UF (1.08 ± 0.93), 67.5% in healthy myometrium (1.40 ± 1.18); Predominant: PE, PP, PS, PA; PE exposure significantly increases UF risk and positively correlates with UF size.
Zhang et al., 2025^31^	China	48 endometrium	Raman, Py-GC/MS	100% detection; Predominant: PTFE, PS, PC, PE, PVC; Py-GC/MS revealed polymer co-occurrences undetected by Raman alone.
Si et al. 2026^32^	China	220 FF (110 DOR, 110 Ctrl)	Py-GC/MS, LDIR	100% detection; Predominant: PA66, PVC, PS; MP levels significantly higher in patients with DOR; PA66 showed the strongest association with DOR risk.
Zhou et al. 2026^33^	China	45 FF (25 DOR, 20 Ctrl)	Py-GC/MS, LDIR, SEM	100% detection (DOR: 30.63 μg/g vs. Ctrl: 18.48 μg/g); Predominant: PE, PP, PVC; Total MPs negatively correlated with AMH levels and AFC.

MPs, microplastics; AFC, Antral follicle count; AMH, Anti-Müllerian hormone; Ctrl, Control; DOR, Diminished ovarian reserve; FF, Follicular fluid; FSH, Follicle-stimulating hormone; IVF, In vitro fertilisation; MPs, Microplastics; UF, Uterine fibroids; FTIR, Fourier-transform infrared spectroscopy; LDIR, Laser direct infrared imaging; Py-GC/MS, Pyrolysis-gas chromatography/mass spectrometry; SEM, Scanning electron microscopy; SEM-EDX, Scanning electron microscopy with energy dispersive X-ray spectroscopy; O-PTIR, Optical photothermal infrared; ACR, Acrylic resin; BR, Butadiene rubber; CPE, Chlorinated polyethylene; EVA, Ethylene-vinyl acetate; PA, Polyamide; PA66, Polyamide 66; PBAT, Polybutylene adipate terephthalate; PC, Polycarbonate; PE, Polyethylene; PE-co-PP, Polyethylene-polypropylene copolymer; PET, Polyethylene terephthalate; PMMA, Polymethyl methacrylate; PP, Polypropylene; PS, Polystyrene; PTFE, Polytetrafluoroethylene; PU, Polyurethane; PVC, Polyvinyl chloride.

The average concentrations and compositions of MPs varied significantly across different tissues as well as within the same tissue types. Multiple factors can explain these discrepancies, with different detection methodologies possessing inherent limitations. For example, LDIR is a commonly used technique characterised by rapid imaging and the ability to resolve particle shapes. However, it cannot detect particles smaller than 20 μm,^25^ which leads to an underestimation of MP content and restricts the analysis to particle counting alone. Conversely, Py-GC/MS achieves absolute quantification through high-temperature thermal degradation and separation. This method offers high sensitivity and specificity but lacks sufficient resolution between different components. Consequently, this deficiency can result in misjudgements, including false positive results where lipids are incorrectly identified as MPs.^34^ Furthermore, population heterogeneity likely contributes to the observed variations among studies. Due to the high costs associated with detection technologies, current research features small sample sizes that are predominantly drawn from single geographic regions. Significant differences exist among diverse populations regarding race, living environments, lifestyles, and dietary habits. The patient cohorts also present diverse clinical conditions, such as recurrent spontaneous abortion^13^ and uterine fibroids,^30^ as well as in healthy populations.^25^ High risk factors for MP exposure among patients, such as prior surgeries or smoking habits, are difficult to standardise, complicating efforts to ensure methodological consistency. The following sections will detail the specific impacts of MPs on the distinct reproductive systems of men and women.

### Impact on sperm quality and the male reproductive system

Over the past two centuries, male sperm quality and quantity have consistently declined.^35^^,^^36^ Among populations with infertility, male factors represent the primary or contributing cause in approximately 50% of cases.^37^ Consequently, investigating the potential risk factors associated with declining male fertility is critical for advancing reproductive health; such insights could potentially reduce the reliance on ART for nearly half of affected couples.

To date, only two studies have reported the presence of MPs in human testicular tissue.^15^^,^^17^ For instance, Hu et al. discovered a negative correlation between polyvinyl chloride and polyethylene terephthalate concentrations and the normalised weight of the testis.^17^ Given the relative accessibility of semen samples and the extensive information they provide regarding male reproductive function, semen quality is increasingly recognised as a sensitive indicator of environmental and human health. Environmental pollutants can potentially affect sperm chromatin by oxidative stress, alter sperm methylation, and impair DNA binding.^38^, ^39^, ^40^, ^41^ MPs may plausibly induce sperm chromatin instability through similar pathways, although direct mechanistic evidence in humans remains limited.^42^ The detection and characterisation of MPs in human semen are therefore important for assessing exposure within the male reproductive system and investigating its potential reproductive consequences. A study by Ren et al. demonstrated that MP concentrations in patients with asthenozoospermia were significantly higher than those in normal controls,^23^ providing strong evidence for an association between MPs and male reproductive toxicity. Furthermore, several studies have indicated that specific MP compositions, including polytetrafluoroethylene, polyethylene terephthalate, polystyrene, and polyvinyl chloride, are associated with decreased total sperm count, sperm concentration, and progressive motility.^18^, ^19^, ^20^, ^21^^,^^43^ However, the sample sizes in these existing studies remain limited, necessitating larger scale, broader, and multicentre collaborative research.

Spermatogenesis is a relatively dynamic process. Whether the accumulation of MPs in semen is short-term, potentially involving clearance mechanisms, or constitutes long-term retention requires a dynamic understanding. Clinically, semen analysis is an economical and non-invasive cornerstone for assessing male fertility during ART evaluations. However, test results are frequently variable, necessitating repeated assessments. Therefore, future studies investigating the relationship between MPs and sperm parameters must incorporate longitudinal semen analyses to elucidate the definitive impact of these particles.

Given the routine collection of semen in ART populations, future research should investigate whether the accumulation levels and physical characteristics of MPs in sperm can serve as predictive biomarkers for embryo yield, overall fertility outcomes, and subsequent offspring health. Additionally, integrating MP profiling with other readily accessible clinical indicators to develop high-accuracy predictive models could offer novel strategies for personalised, precision ART treatments.

### Impact on ovarian reserve, endometrial receptivity, and the female reproductive system

Compared to male reproductive physiology, female reproductive physiology is considerably more complex. Women possess a finite ovarian reserve determined by the number of primordial follicles present at birth. Oocyte development is strictly regulated by hormonal fluctuations during the menstrual cycle; under physiological conditions, only one or two mature oocytes are ovulated per cycle. Furthermore, oocyte quality declines significantly after the age of 35. These physiological constraints frequently manifest as clinical challenges for women with infertility, presenting as diminished ovarian reserve (DOR), primary ovarian insufficiency (POI), poor ovarian response (POR), and the generation of aneuploid embryos. Previous studies support that MP levels in FF are positively correlated with follicle stimulating hormone^27^ and negatively correlated with antral follicle count,^33^ while their relationship with anti-Müllerian hormone remains controversial.^27^^,^^33^ This evidence suggests that MP exposure in FF may be associated with DOR in women. More directly, a recent study revealed that MP concentrations were significantly higher in patients with DOR, where polyamide 66 showed the strongest association with this risk.^32^ Furthermore, several studies have reported lower fertilisation rates with higher MP exposure.^21^^,^^29^ Therefore, the accumulation of MPs in FF may pose harmful effects on the quantity and quality of oocytes, thereby profoundly impacting female reproductive health and reproductive lifespan.

Oocytes serve as the foundational seeds of life. While rapid advancements in ART, such as controlled ovarian hyperstimulation, in vitro fertilisation (IVF), and preimplantation genetic testing (PGT), can largely secure oocyte quantity and quality, the endometrium, acting as the soil for embryonic development, remains a “black box” in ART. Current understanding of the establishment and maintenance of pregnancy remains insufficient. Despite the transfer of euploid blastocysts across multiple IVF-cycles, the success rate hovers at approximately 65%.^44^ Recurrent implantation failure (RIF), recurrent pregnancy loss, and unexplained infertility are all closely associated with endometrial receptivity (ER). Numerous studies have indicated the presence of MPs in the endometrium,^13^^,^^25^^,^^26^^,^^30^^,^^45^ which provides valuable insights into unexplained infertility. Although direct evidence linking MPs to these specific conditions is still emerging, existing data strongly suggest that MPs represent a critical risk factor for decreased ER, thereby establishing a clear direction for future research. Additionally, MP abundance was found to be significantly higher in endometrial polyps compared to normal endometrium,^26^ and polyethylene exposure significantly increases the risk of uterine fibroids.^30^ These findings collectively indicate that MPs may induce structural and functional abnormalities in the uterus, providing crucial clues for further investigating their impact on reproductive health.

Future studies should thoroughly investigate a broader spectrum of reproductive diseases, including premature ovarian failure, polycystic ovary syndrome, and other ovarian disorders, as well as endometrial pathologies like endometriosis, and pathophysiological changes including fallopian tube adhesions, blockages, and vaginitis. Beyond the direct toxicity caused by MP exposure in the endometrium, research conducted by Sun et al. on mouse models indicates that the gut microbiota may participate in the process through which MPs reduce ER.^46^ This highlights that future research must focus on multiorgan interactions, such as the relationship between the vaginal microbiota and ER, the influence of MPs on the hypothalamic pituitary ovarian axis, and their broader neuroendocrine impacts.

Concurrently, integrating these foundational insights into clinical practice is essential for optimising ART outcomes. Recognising the profound role of environmental disruptors, specifically MP exposure, offers novel, preventative strategies for fertility management. On a clinical level, this necessitates enhancing patient counselling during preconception care. By actively educating patients to minimise their daily exposure to everyday plastic products, such as plastic water bottles, food containers, and cosmetics, clinicians can empower patients to proactively mitigate potential reproductive toxicity before initiating ART.

## Concerns for MP exposure during ART procedures

Particularly crucial is the heightened attention required for the health risks posed by MPs in ART for couples with infertility, especially when contrasted with the natural reproductive process. ART encompasses a series of intricate procedures aimed at achieving pregnancy, including but not limited to artificial insemination, oocyte retrieval, IVF, and embryo culture.^47^ Throughout these procedures, gametes, embryos and reproductive organs are frequently in contact with various plastic devices. For instance, during artificial insemination and sperm retrieval, plastic containers and collection tubes are routinely used. Oocyte retrieval is typically performed using plastic needles and aspiration tubes to collect eggs from the ovaries. Subsequently, fertilisation and embryo culture are performed in plastic dishes, and diagnostic procedures like hysteroscopy employ plastic reservoirs for uterine distension fluids. The pervasive use of these consumables raises critical concerns regarding the vulnerability of gametes and embryos to plastic-derived contaminants.

Moreover, because invasive medical procedures provide direct pathways for MP introduction,^48^ the surgical components of ART may inherently cause iatrogenic contamination. Experimental simulations of routine infusion and blood-collection procedures have quantified MP release from infusion tubing and blood-collection needles.^49^ Although direct clinical evidence of device-derived MP exposure during routine medical care remains limited, increased blood MP concentrations have been reported after percutaneous coronary intervention, with the detected polymer profiles resembling those identified in device-rinse samples.^50^ These findings raise the possibility of procedure-related intravascular contamination. Collectively, the available evidence suggests that iatrogenic MP exposure during ART is biologically plausible but remains insufficiently characterised. Therefore, understanding the interactions between reproductive cells and plastic devices is crucial for optimising ART outcomes. To date, few studies have investigated the impact of MPs on assisted reproduction and the health outcomes of infants conceived via IVF. Significant knowledge gaps remain regarding the specific characteristics of MP exposure during ART, including polymer composition, particle size, and morphology. Furthermore, it remains definitively unclear whether this exposure adversely affects embryo quality, implantation rates, and overall live birth rates. Additionally, the potential contribution of MP exposure to pregnancy complications and adverse perinatal outcomes must not be overlooked.

## Maternal-foetal transfer, early-life exposure, and transgenerational risks

Parental exposure to environmental factors may trigger transgenerational epigenetic inheritance that impacts fertility by affecting gonadal function. In animal models, parental or gestational exposure to MPs has been associated with altered DNA methylation and histone profiles in offspring testes.^51^ MPs may also impair the development and function of the nervous, immune, and reproductive systems in offspring through inflammation, oxidative stress, and endocrine-disrupting effects.^52^^,^^53^ To characterise human exposure at the maternal–foetal interface, we conducted a comprehensive literature search using the terms “microplastics”, “nanoplastics”, “placenta”, “amniotic fluid”, “cord blood”, and “meconium”. All studies reporting the presence of MPs in human maternal-foetal compartments are summarised in Table 2. The majority of these studies were also conducted in China (17/27), although the regions and populations investigated were more diverse than those in studies focusing solely on the reproductive system. Furthermore, the sample sizes were substantially larger, reaching up to 1750 participants, which underscores the profound importance of investigating the potential toxicity of MPs to the fetus. Detection rates ranged from 62 to 100% in placentas, 66.7–100% in amniotic fluid (AF), 55.6–100% in cord blood, 0–100% in meconium, and 100% in infant faeces. The variance among these detection rates could be explained by the fact that the blood placental barrier only permits the passage of MPs with smaller particle sizes. Consequently, the MPs detectable by FTIR and LDIR are extremely limited. Additionally, placental detection results from different time periods suggest that MP abundance in placentas has steadily increased over recent years.^61^ Moreover, in addition to the physiological reasons listed above, meconium is highly susceptible to contamination from urine and diapers during the sample collection process, which may also contribute significantly to the observed discrepancies in detection rates.

**Table 2:** Characteristics and main findings of studies detecting MPs in human maternal-foetal compartments.

Study	Location	Samples	Detection methods	Main findings
Placenta				
Braun et al., 2021^54^	Germany	Placenta, meconium	FTIR	Screened positive; Predominant: PE, PP, PS, PU; PU also detected as airborne fallout in operating room (potential contamination source).
Ragusa et al., 2021^55^	Italy	6 placentas	Raman	66.7% detection; Found on foetal, maternal, and chorioamniotic membrane sides.
Amereh et al., 2022^56^	Iran	43 placentas (13 IUGR, 30 Ctrl)	Raman	100% detection in IUGR vs. 13% in controls; Predominant: PE, PS; Inverse association with birth weight and 1-min Apgar score in IUGR cases.
Ragusa et al., 2022^57^	Italy	8 placentas	VP-SEM, TEM	Detected intracellularly (carbon-rich fragments); Localised in syncytiotrophoblast; Correlated with ultrastructural alterations of mitochondria and endoplasmic reticulum.
Halfar et al., 2023^58^	Czech Republic	10 placentas, AF	FTIR	90% detection; Predominant: CPE, calcium zinc PVC stabilisers.
Liu et al., 2023^59^	China	18 placentas, 12 meconium	LDIR	100% detection (median: 18.0, 51.4 particles/g in placentas, and meconium respectively); Predominant: PA, PU; High MP concentrations linked to significant shifts in placental and meconium microbiota diversity.
Liu et al., 2023^60^	China	18 placentas, 12 meconium, 12 infant faeces	LDIR	100% detection (median: 18.0, 51.4, 26.6 particles/g in placentas, meconium, and infant faeces, respectively); Predominant: PA, PU.
Weingrill et al. 2023^61^	USA	30 placentas	Raman	Detection increased over time (60% in 2006 to 100% in 2021); Predominant: PP, PES, PET, PVA; MPs abundance and size increased significantly across years.
Zhu et al., 2023^62^	China	17 placentas	LDIR	100% detection (mean 2.70 particles/g); Predominant: PVC (43.3%), PP, PBS.
Garcia et al., 2024^63^	Mexico	64 placentas	Py-GC/MS, FTIR	100% detection (mean 126.8 μg/g); Predominant: PE (54%), PVC, nylon.
Sun et al., 2024^64^	China	12 placentas, AF, foetal membrane, umbilical vein blood, umbilical cord	LDIR	Detected across 6 compartments (median particles/g): umbilical cord (10.40), maternal blood (8.18), foetal membrane (6.56), AF (4.80), placenta (4.68), and umbilical vein blood (2.73).; Predominant: PA, PU; MPs abundance in AF positively correlated with maternal age and pre-pregnancy BMI.
Yun et al., 2024^65^	China	50 placentas	Raman	62% detection; Predominant: PTFE, PS, ABS; No significant association with immediate placental pathological alterations.
Zhu et al., 2024^66^	China	9 placentas, cord blood, meconium	Raman	Detection rates: placenta (100%, median 3.15 particles/g), cord blood (55.6%, 1.1 particles/g), meconium (100%, 20.75 particles/g); Predominant: CEL (placenta), PB (cord blood), PE (meconium); Lower meconium MPs in women drinking tea ≥3 times/week.
Liu et al., 2025^67^	China	1324 placentas	LC-MS/MS, LDIR	100% detection (median: 1.2 particles/g); Predominant: PVC, PBS; Linked to endocrine disruption (lower foetal cortisol, altered glucocorticoid/androgenic ratios).
Oliveira et al., 2025^68^	Brazil	10 placentas, umbilical cord	Raman	Detected 110 and 119 particles in placentas and umbilical cords, respectively; Predominant: PE, PA, PEVA, PU, PP.
Wang et al., 2025^69^	China	1057 placentas	LDIR	Median: 1.2 particles/g; Predominant: PVC, PP, PBS; Strongly associated with elevated maternal liver enzymes (ALP, ALT, AST, GGT).
Yin et al., 2025^70^	China	1350 placentas	LDIR	100% detection (median: 0.7 particles/g); Predominant: PVC, PBS, PP; Associated with elevated maternal creatinine and cystatin C (indicating potential renal impairment).
Zhang et al., 2025^71^	China	1121 placentas	LDIR	Median 1.5 particles/g; Predominant: PVC, PP, PBS; Higher PVC/PBS levels associated with significantly reduced cord blood telomere length.
Zhang et al., 2025^72^	China	1250 placentas	LDIR	Mean: 1.084 particles/g; Predominant: PVC, PP, PBS; Associated with thyroid disruption (reduced foetal T4, TSH levels, and T4/T3 ratio).
Shen et al., 2026^73^	China	1750 placentas	LDIR	Median: 2 particles/g; Predominant polymers: PVC, PP, PBS, PET; Total MPs strongly linked to reduced birth weight, length, and head circumference (stronger effects in males).
AF				
Xue et al., 2024^74^	China	40 AF	LDIR	80% detection (mean 2.01 particles/g); Predominant: PE, CPE; Positively correlated with maternal seafood/bottled water intake; negatively with gestational age.
Tian et al., 2025^75^	China	39 AF	Raman, Py-GC/MS	6 distinct polymers detected; Predominant: PTFE, PS, ABS; No significant associations with immediate adverse pregnancy outcomes.
Sharma et al. 2026^76^	India	5 AF, 3 menstrual blood	FTIR, Raman, Py-GC/MS, AFM	100% detection (37 particles in AF); Predominant: PS, PC.
Meconium				
Li et al., 2023^77^	China	16 meconium	FTIR	No MPs detected.
Li et al., 2025^78^	China	60 meconium	Py-GC/MS	51.67% to 71.67% detection rates (1.60∗10 = 5 to 1.53∗10-3 μg/g); Predominant: PE, ABS, PVC.
Others				
Bissonnette et al., 2025^79^	Canada	46 placental blood	Py-GCxcIMS	Detected in 31% of replicates (mean: 0.9 μg/mL); Predominant: PP, PE, PS.
Yin et al., 2025^80^	China	10 cord blood	Py-GC/MS, SEM	100% detection (mean 41.128 μg/g); Predominant: PS, PE, PVC, PP.

MPs, microplastics; AF, Amniotic fluid; ALP, Alkaline phosphatase; ALT, Alanine aminotransferase; AST, Aspartate aminotransferase; BMI, Body mass index; Ctrl, Control; GGT, Gamma-glutamyl transferase; IUGR, Intrauterine growth restriction; MPs, Microplastics; T3, Triiodothyronine; T4, Thyroxine; TSH, Thyroid-stimulating hormone; FTIR, Fourier-transform infrared spectroscopy; LC-MS/MS, Liquid chromatography-tandem mass spectrometry; LDIR, Laser direct infrared imaging; Py-GC/MS, Pyrolysis-gas chromatography/mass spectrometry; Py-GCxcIMS, Pyrolysis-comprehensive gas chromatography-ion mobility spectrometry; Raman, Raman spectroscopy; AFM, Atomic force microscopy; SEM, Scanning electron microscopy; TEM, Transmission electron microscopy; VP-SEM, Variable pressure scanning electron microscopy; ABS, Acrylonitrile butadiene styrene; CEL, Cellulose; CPE, Chlorinated polyethylene; PA, Polyamide; PB, Polybutene; PBS, Polybutylene succinate; PC, Polycarbonate; PE, Polyethylene; PES, Polyethersulfone; PET, Polyethylene terephthalate; PEVA, Polyethylene vinyl acetate; PP, Polypropylene; PS, Polystyrene; PTFE, Polytetrafluoroethylene; PU, Polyurethane; PVA, Polyvinyl alcohol; PVC, Polyvinyl chloride.

As a critical barrier for material exchange and communication between the mother and the fetus, the placenta has been shown in multiple studies to accumulate MPs.^54^, ^55^, ^56^, ^57^, ^58^, ^59^, ^60^, ^61^, ^62^, ^63^^,^^65^, ^66^, ^67^, ^68^, ^69^, ^70^, ^71^^,^^73^^,^^81^ At the tissue level, pathological alterations such as villous infarction, fibrin deposition, syncytial knots, villous maturation, and inflammatory infiltrates occur, with no significant differences observed among various MP polymers.^65^ However, at the subcellular level, the presence of MPs is possibly correlated with ultrastructural alterations of the mitochondria and endoplasmic reticulum.^57^ Results from large cohort studies (n > 1000) indicate that, for the mother, MP exposure is associated with elevated maternal creatinine and cystatin C,^70^ as well as elevated maternal alkaline phosphatase, alanine aminotransferase, aspartate aminotransferase, and gamma glutamyl transferase.^69^ These elevations pose significant threats to the kidneys and liver, which are vital and highly susceptible organs during pregnancy. For the fetus, the concentration of MPs in placental tissue may lead to foetal growth restriction, specifically manifesting as reduced birth weight, length, and head circumference.^56^^,^^73^ Utilising Raman spectroscopy detection techniques, researchers observed a 100% detection rate in intrauterine growth restriction cases vs. 13% in healthy controls, although this specific finding was not from a large sample study.^56^ Concurrently, the MP content in the placenta correlated with a reduced 1-min Apgar score for newborns,^56^ indicating that MP exposure in the placenta significantly increases the risk of neonatal asphyxia. Furthermore, large sample studies indicate that MPs are linked to endocrine disruption, associated with lower foetal cortisol, altered glucocorticoid to androgenic ratios,^67^ and reduced foetal thyroxine, thyroid stimulating hormone levels, and thyroxine to triiodothyronine ratio,^72^ which are essential hormones for proper foetal development. Additionally, a study by Zhang et al. revealed an association between higher polyvinyl chloride and polybutylene succinate levels and reduced cord blood telomere length,^71^ indicating that prenatal exposure to MPs may play a detrimental role in child health over the long term.

AF, primarily derived from cord blood and foetal metabolites, also reflects foetal MP exposure within the uterine environment. Multiple studies have reported the continuous presence of MPs in AF.^58^^,^^64^^,^^74^, ^75^, ^76^ A study by Tian et al. reported no significant differences in gestational duration, gestational diabetes mellitus incidence, newborn length, weight, and sex associated with MP exposure.^75^ Conversely, research by Xue et al. indicated a negative correlation between MP abundance in AF and gestational age.^74^ Because research regarding the exact association between MPs, pregnancy complications, and birth outcomes remains somewhat contradictory and limited, further robust investigation is urgently needed.

Finally, a study by Liu et al. indicated that high MP concentrations are linked to significant shifts in placental and meconium microbiota diversity,^59^ which may cause compromised intestinal barrier function and increase susceptibility to infections.^82^ In addition, the presence of MPs has been detected in various other critical tissues and biological samples, including placental blood, infant faeces, foetal membranes, and cord blood.^60^^,^^64^^,^^66^^,^^68^^,^^79^ Additional research is required to definitively correlate the accumulation of MPs in these specific tissue samples with broader clinical outcomes.

## Implications, limitations, and future perspectives

The pervasive infiltration of MPs into the human body has emerged as a formidable threat to global public health, with reproductive systems being particularly vulnerable. Current evidence demonstrates the presence of MPs across male and female reproductive systems, as well as within placental and foetal tissues. These ubiquitous particles are associated with DOR, impaired sperm quality, and adverse pregnancy outcomes. However, these associations should be interpreted with caution because most available studies are observational, and causality has not yet been established.

In the context of ART, the key concern is that it may represent a distinct and biologically sensitive exposure window for MP exposure. Throughout the intricate clinical and laboratory procedures of assisted reproduction, vulnerable gametes and embryos are placed in direct and prolonged contact with a multitude of plastic medical devices and culture environments. Whether this concentrated exposure carries profound risks of compromising embryo developmental potential, exacerbating RIF, and inflicting transgenerational epigenetic harm on the resulting offspring remains to be further investigated. Nevertheless, direct evidence demonstrating that ART procedures increase MP exposure or compromise clinical outcomes remains scarce. Therefore, the current evidence does not justify advising people to delay or forgo ART solely because of concerns about MPs. Instead, these uncertainties highlight the need for balanced counselling, rigorous laboratory quality control, and targeted research.

Future studies should prioritise large-scale, prospective, multicentre cohorts to estimate the association between MP exposure and human fertility, ART outcomes, pregnancy complications, and offspring health. Such studies should integrate MP measurements in reproductive samples with clinically meaningful outcomes such as oocyte yield, fertilisation rate, embryo development, implantation, miscarriage, livebirth, and perinatal health. Experimental studies under clinically relevant ART conditions are also needed to evaluate whether commonly used consumables, including dishes, tubes, pipette tips, catheters, cryopreservation devices, and culture media packaging, release MPs or nanoplastics during routine use. In parallel, the reproductive medicine community must critically re-evaluate the ubiquitous reliance on plastic consumables within embryology laboratories. Developing safer, MP-free alternative materials for gamete handling and embryo culture is a pressing necessity. Furthermore, establishing longitudinal tracking cohorts for children conceived via ART is absolutely essential to monitor potential long-term developmental and neuroendocrine consequences. Given that early-life environmental exposures may influence developmental trajectories partly through epigenetic regulation,^83^ future cohorts should consider integrating epigenomic, endocrine, metabolic, and neurodevelopmental assessments where feasible.

Several limitations of the current evidence should be recognised. First, most human studies are limited by small sample sizes, single-centre recruitment, and heterogeneous populations, which restricts the generalisability of findings. This limitation is particularly important for studies linking MP distribution with clinical outcomes, because MP exposure is likely to vary according to geography, occupation, diet, lifestyle, socioeconomic factors, medical history, and laboratory practice. Second, many studies are cross-sectional and cannot distinguish whether MPs contribute to reproductive dysfunction, accumulate as a consequence of altered tissue physiology, or merely reflect background environmental exposure. Third, the effects attributable to plastic particles themselves remain difficult to disentangle from those of plastic-associated chemicals and co-contaminants. As derivatives of commercial plastic products, MPs may contain or leach manufacturing additives, including plasticisers, bisphenols, stabilisers, flame retardants, pigments, fillers, antioxidants, and surfactants.^4^ After environmental ageing, their large specific surface area and surface charge also enable adsorption of persistent organic pollutants, per- and polyfluoroalkyl substances, phthalate esters, heavy metals, pathogens, and antibiotic-resistance genes.^10^^,^^11^ Therefore, the biological effects associated with MPs are unlikely to be determined solely by particle size, morphology, polymer composition, surface charge, or functional groups, but may also reflect the combined or synergistic effects of associated chemical and biological hazards. This complexity complicates causal attribution in human studies and should be explicitly considered in future toxicological and epidemiological research.

Methodological heterogeneity is another major challenge. Many studies rely on a single detection method, yet each analytical platform has intrinsic limitations. Py-GC/MS provides polymer-specific mass-based quantification, but it requires thermal destruction of samples and cannot characterise particle size, morphology, or tissue localisation. Conversely, imaging-based techniques such as LDIR, FTIR, and Raman spectroscopy can provide information on particle number, shape, and spatial distribution, but their resolution, throughput, polymer identification accuracy, and ability to detect nanoplastics remain limited. Future investigations should therefore consider orthogonal analytical strategies that combine mass-based quantification with particle imaging and chemical confirmation, thereby providing a more comprehensive assessment of MP abundance, polymer composition, size distribution, morphology, and localisation.

Environmental contamination during MP measurement remains a major source of uncertainty, particularly when particle concentrations are low or when small particles are targeted. Airborne fallout, laboratory dust, plastic consumables, filtration devices, sample containers, aluminium foil, solvents, and digestion or flotation reagents can introduce exogenous particles during sampling, pretreatment, filtration, and spectroscopic analysis.^84^^,^^85^ Importantly, clean filters alone are insufficient as blank controls because they do not account for contamination introduced across the complete analytical workflow. Representative procedural blanks that undergo all handling, pretreatment, filtration, and analytical steps are therefore essential for estimating the limit of blank, limit of detection, and limit of quantification, and for performing blank-corrected particle counts.^86^ Reagent-derived contamination also requires particular attention because commonly used flotation and digestion solutions may contain MPs, many of which are smaller than 100 μm, and may therefore lead to overestimation of sample abundance.^87^ Future studies should incorporate contamination-control procedures at the design stage, including procedural blanks, airborne fallout controls, pre-filtered reagents, non-plastic or low-shedding equipment, covered sample processing, recovery experiments, and transparent reporting of blank correction.

This personal view also has limitations. First, we focused primarily on the distribution of MPs in human reproductive and maternal-foetal tissues and their associations with clinical outcomes. We did not comprehensively review the dynamic processes by which MPs enter the human body, cross biological barriers, accumulate in tissues, undergo clearance, or interact with metabolic and excretory pathways. Such dynamic processes may be particularly relevant to reproductive organs and biological fluids with cyclical or time-dependent characteristics, such as spermatogenesis, follicular development, endometrial remodelling, and menstruation. Second, our discussion mainly centred on reproductive tissues and reproductive-related biological samples. However, MPs have also been detected in non-reproductive samples, including urine, and urinary MP burden has been associated with gestational diabetes mellitus in a recent study.^88^ These findings suggest that MP exposure may reflect broader systemic distribution and may be linked to metabolic or inflammatory disturbances that could indirectly affect reproductive health and pregnancy outcomes. Future studies should therefore evaluate reproductive MP exposure within a whole-body framework. Third, this personal view was not intended to provide a detailed mechanistic review of MP reproductive toxicity. Although potential pathways are briefly discussed where relevant, most mechanistic evidence remains derived from animal or in vitro models and was not systematically evaluated here. Emerging intervention studies also warrant attention. Experimental studies have suggested that anthocyanins and probiotics may attenuate MP-induced toxicity.^89^^,^^90^ More importantly, a recent two-phase population study reported that composite polyphenol supplementation may mitigate the pro-inflammatory effects associated with high MP exposure.^91^ Therefore, future research determining whether feasible interventions can reduce MP-associated reproductive risks in humans is expected.

## Conclusions

Current evidence indicates that MPs are present in human reproductive and maternal–foetal tissues and may be associated with impaired reproductive function, pregnancy-related complications, and early-life exposure, although causality remains unproven. For people undergoing ART, contact between gametes, embryos, and plastic materials represents a plausible but insufficiently characterised exposure pathway. Future studies should integrate standardised MP assessment with clinically relevant ART, pregnancy, and offspring outcomes.

## Outstanding questions

To fully elucidate and effectively mitigate the potential risks of microplastic contamination, the field will require a coordinated effort encompassing standardised detection protocols, rigorous contamination controls, prospective multicentre cohort studies, and the adoption of safer laboratory materials, all of which are essential for generating reliable evidence and guiding evidence-based practice.

**Search strategy and selection criteria**

A comprehensive literature search was conducted using the following terms: “microplastics”, “nanoplastics”, “assisted reproductive technology”, “in vitro fertilisation”, “intracytoplasmic sperm injection”, “embryo culture”, “oocyte retrieval”, “embryo transfer”, “semen”, “follicular fluid”, “testis”, “ovary”, “endometrium”, “uterine”, “placenta”, “amniotic fluid”, “cord blood”, “meconium”, “medical device”, “syringe”, and “catheter”. No restrictions were applied regarding publication year or language. Ultimately, only studies specifically investigating the presence of microplastics in human tissues and biological samples were included in this evaluation.

## Contributors

XQ conceptualised the paper. DM was responsible for writing—original draft. WN was responsible for visualisation. JL and BS conducted the literature search. DM, RL, KLH, and XQ acquired funding for this work. RL, KLH, and XQ were responsible for writing – review & editing. DM and WN have directly accessed and verified the underlying data reported in the manuscript. All authors read and approved the final version of the manuscript.

## Declaration of interests

The authors declare that they have no competing interests.

## Figures and Tables

**Graphical abstract:**
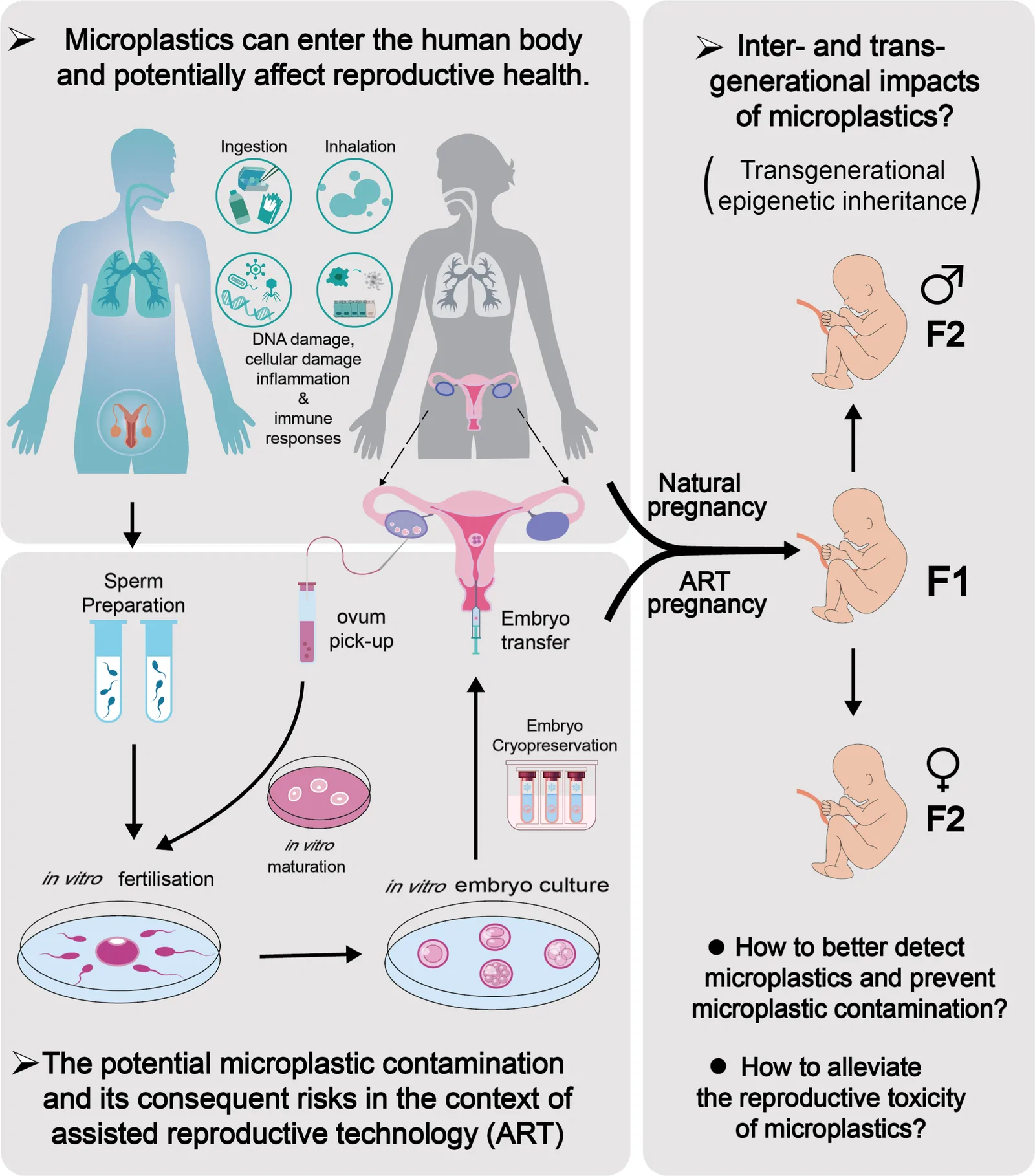
What are the effects of microplastics on human reproductive health? Microplastics can invade the reproductive system tissues and organs through ingestion, inhalation, and even through the reproductive tract, potentially impacting reproductive health. However, the effects of microplastics on this system remain unclear, particularly concerning assisted reproductive health in patients with infertility, which may pose or exacerbate reproductive health risks. Additionally, the possibility of transgenerational epigenetic effects should not be overlooked.
